# Performance Measurement of Gesture-Based Human–Machine Interfaces Within eXtended Reality Head-Mounted Displays

**DOI:** 10.3390/s25092831

**Published:** 2025-04-30

**Authors:** Leopoldo Angrisani, Mauro D’Arco, Egidio De Benedetto, Luigi Duraccio, Fabrizio Lo Regio, Michele Sansone, Annarita Tedesco

**Affiliations:** 1 Department of Electrical Engineering and Information Technology (DIETI), University of Naples Federico II, 80125 Napoli, Italy; leopoldo.angrisani@unina.it (L.A.); darco@unina.it (M.D.); luigi.duraccio@unina.it (L.D.); fabrizio.loregio@unina.it (F.L.R.); 2 Department of Precision and Regenerative Medicine and Ionian Area-Section of Engineering, University of Bari Aldo Moro, 70121 Bari, Italy; m.sansone24@phd.uniba.it; 3 Department of Public Health, University of Naples Federico II, 80131 Napoli, Italy; annarita.tedesco@unina.it

**Keywords:** eXtended reality, gesture recognition, GUM, human–machine interaction, measurement, metrology, performance characterization, uncertainty

## Abstract

This paper proposes a method for measuring the performance of Human–Machine Interfaces based on hand-gesture recognition, implemented within eXtended Reality Head-Mounted Displays. The proposed method leverages a systematic approach, enabling performance measurement in compliance with the Guide to the Expression of Uncertainty in Measurement. As an initial step, a testbed is developed, comprising a series of icons accommodated within the field of view of the eXtended Reality Head-Mounted Display considered. Each icon must be selected through a cue-guided task using the hand gestures under evaluation. Multiple selection cycles involving different individuals are conducted to derive suitable performance metrics. These metrics are derived considering the specific parameters characterizing the hand gestures, as well as the uncertainty contributions arising from intra- and inter-individual variability in the measured quantity values. As a case study, the eXtended Reality Head-Mounted Display Microsoft HoloLens 2 and the finger-tapping gesture were investigated. Without compromising generality, the obtained results show that the proposed method can provide valuable insights into performance trends across individuals and gesture parameters. Moreover, the statistical analyses employed can determine whether increased individual familiarity with the Human–Machine Interface results in faster task completion without a corresponding decrease in accuracy. Overall, the proposed method provides a comprehensive framework for evaluating the compliance of hand-gesture-based Human–Machine Interfaces with target performance specifications related to specific application contexts.

## 1. Introduction

Human–Machine Interfaces (HMIs) integrate hardware and software systems, enabling a direct communication path between individuals and external devices [1]. HMIs can be classified into two types: Tangible HMIs (THMIs), where interaction occurs through physical devices (e.g., keyboards and mice) that users must handle [2], and Natural HMIs (NHMIs), where interaction occurs through users’ actions (e.g., movements of the eyes or hands, as well as vocal commands) recognized by the interface [3]. Although NHMIs offer more flexible and intuitive interaction compared to THMIs [4], the transition from THMIs to NHMIs has occurred only in recent years, driven by the development of enabling technologies within the *Industry 4.0* landscape, such as Artificial Intelligence (AI), Internet of Things (IoT), and eXtended Reality (XR) [5,6]. A common example of an NHMI is a voice assistant, which enables interaction through natural language, facilitating intuitive and efficient communication [7]. With specific regard to XR [8,9], the use of Head-Mounted Displays (HMDs) has allowed the development of more immersive environments that have enabled seamless interaction, blending the physical and digital realms [10].

Among the different ways of interaction, NHMIs based on hand-gesture recognition have garnered considerable attention for their ability to translate hand movements into digital information [11]. In fact, the implementation of these interfaces within XR HMDs has broadened their application in diverse domains, including entertainment [12], healthcare [13], and industry [14]. However, despite the increasing interest, comprehensive specifications are still lacking regarding their performance, as well as methods for deriving such information. In particular, XR manufacturers typically provide only general information about the types of sensor systems integrated into their devices, such as visible light cameras, infrared (IR) cameras, LIDAR, depth cameras, and inertial measurement units (IMUs), without offering detailed technical specifications. Furthermore, due to the limited information available about the processing strategies responsible for translating the collected data (i.e., hand movements) into digital information, assessing whether a given XR HMD allows the implementation of gesture-based NHMI with adequate performance to justify its suitability for specific application contexts becomes challenging.

Based on these considerations, this paper proposes a novel measurement method for assessing the performance of NHMIs based on hand gesture recognition, implemented within XR HMDs. The proposed method offers a holistic perspective and establishes a structured framework for systematically extracting technical specifications for gesture-based NHMIs, enabling the evaluation of their compliance with the target requirements of the specific application scenarios. It is important to point out that the proposed method is compliant with the Guide to the Expression of Uncertainty in Measurement (GUM), and this guarantees the generalizability and reproducibility of the approach.

The method is as follows. First, a testbed is designed, consisting of a set of icons accommodated within the field of view of the considered XR HMD. These icons must be selected through a cue-guided task using the hand gestures under evaluation. Multiple selection cycles involving different individuals are conducted to derive a set of performance metrics, including

- the Euclidean distance error between the selection points recognized by the NHMI and the reference points within the icons;
- the time required to complete the icon selection;
- the accuracy of icon selection;
- the information transfer rate, which quantifies the amount of information effectively conveyed through the NHMI.

These metrics, although well-established, are strategically obtained while accounting for the parameters characterizing the hand gestures under evaluation, as well as the uncertainty contributions arising from intra- and inter-individual variability. Intra-individual variability refers to differences in gesture execution by the same individual on different selection cycles, whereas inter-individual variability pertains to differences in gesture execution among individuals. Considering all these contributions, the proposed method aims to provide a clearer and more comprehensive understanding of the interface’s ability to recognize gestures under varying conditions, thereby assessing its suitability for specific application contexts, both for naive and experienced users.

The paper is organized as follows. Section 2 provides background on gesture-based NHMIs integrated within XR HMDs. Then, Section 3 describes the proposed method. The case study conducted on HoloLens 2 and the finger-tapping gesture is detailed in Section 4, along with the discussion on the obtained results. Finally, the conclusions are drawn and future work is outlined.

## 2. Background

Hand gestures can be recognized based on the hand configuration, which is defined by the pose of the hand joints, the orientation of the palm, and the spatial relationships between the palm and fingers [15].

Depending on temporal relationships, hand gestures can be classified into two categories: static and dynamic [16]. Static gestures involve stationary hand poses, where the configurations of the hand and fingers remain unchanged over time. In contrast, dynamic gestures are characterized by spatial configurations that evolve over time, following specific trajectories through space. Hence, recognizing dynamic gestures requires tracking both temporal patterns and spatial configurations [17]. This, arguably, entails more stringent requirements compared to static gesture recognition.

Sensing technologies used for gesture recognition can also be broadly classified into two main categories: glove-based and vision-based [18]. Glove-based technology relies on sensors such as wrist-worn IMUs, electromyography (EMG), or piezoelectric sensors, which acquire data directly from the user’s hand [19,20]. Although this technology offers high precision in capturing hand movements, it is often intrusive and presents limitations in daily-use applications due to the need for users to wear specialized equipment [21]. In contrast, vision-based technology is non-intrusive, relying on camera systems such as IR, LIDAR, and depth cameras [22], which capture hand images [23]. Vision-based technology is currently the most widely used approach for hand gesture recognition [24], particularly in XR HMDs, as it leverages the camera systems already integrated into the HMDs, eliminating the need for additional sensors, such as IMUs and EMG devices, which would otherwise need to be worn on the user’s hands. As this work addresses the performance measurement of a gesture-based NHMI implemented within XR HMDs, from this point onward, vision-based technology will be referred to for gesture recognition.

**Figure 1:**
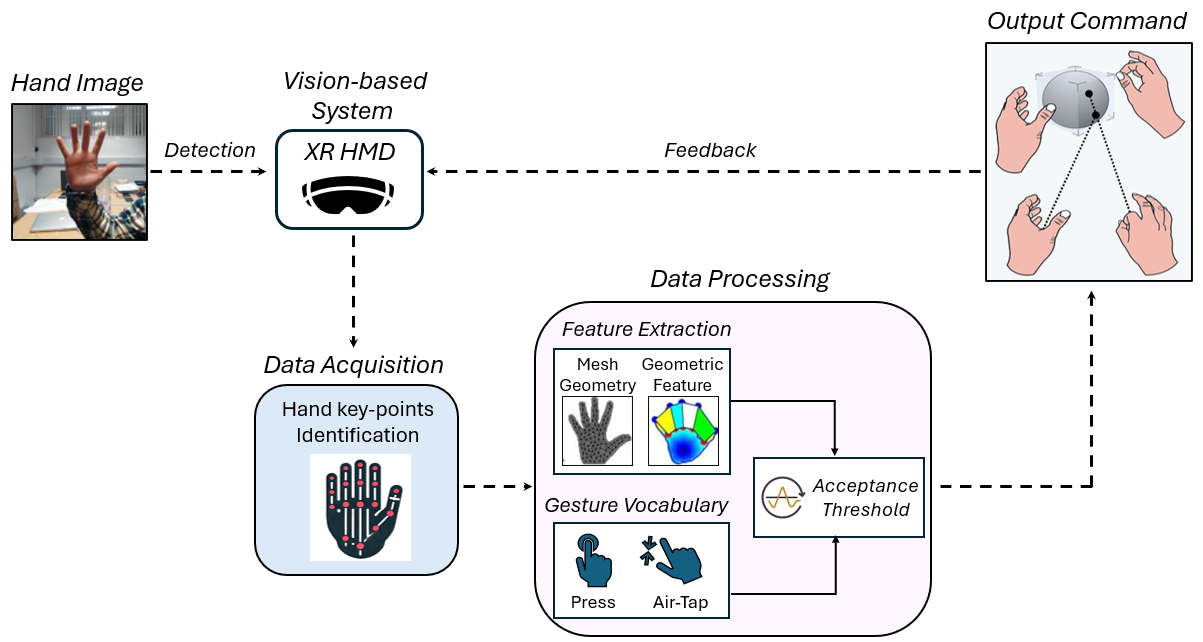
General block diagram of a vision-based hand gesture recognition NHMI implemented within XR HMDs.

In Figure 1, a block diagram of the gesture recognition pipeline in XR HMDs is sketched.

Hand images are captured using a vision-based system, specifically an XR HMD. This system identifies key points within the images, extracts relevant features, and compares them with predefined gesture models that are part of a known vocabulary. To ensure reliable interaction, gesture recognition is typically determined based on acceptance thresholds, which refer to criteria to establish whether a user’s action satisfies the requirements to be identified as a specific gesture [25]. These thresholds are established considering the inherent uncertainty in gesture recognition, which arises from multiple factors:

- Non-ideality of the employed cameras, characterized by technical specifications such as frame rate, pixel resolution, and bit depth per pixel;
- Imperfect definition of biomechanical gesture models, which may introduce inaccuracies when comparing extracted features;
- Intra-individual variability in gesture execution, caused by factors such as fatigue and adaptability [26];
- Inter-individual variability in gesture execution, resulting from differences in hand anatomy, including size, joint flexibility, movement speed, and trajectory [27];
- Involuntary actions and unintentional hand movements, which contribute additional noise to the recognition process [23,28].

Once the gesture is identified, the corresponding output command is executed. Finally, closing the loop, the NHMI provides feedback to the user regarding the recognized gesture.

Although performance assessment in gesture-based NHMIs has been explored in the literature, primarily considering factors such as lighting conditions, complex backgrounds, and physical constraints [29,30], only a limited number of studies focus on XR-based scenarios [31,32], often considering sensor-based recognition systems, both built-in [33] and external [34]. While some of these studies include repeated measures across participants and report metrics that reflect variability (e.g., standard deviation) [35], a methodological approach specifically designed to model, analyze, and integrate both intra- and inter-individual variability within a replicable performance assessment framework would provide a more comprehensive understanding of the implemented NHMI’s performance.

As a result, a gap remains in the literature regarding reliable measurement procedures for evaluating the performance of vision-based gesture recognition NHMIs implemented within XR HMDs. This gap limits direct comparisons of commercially available devices and compromises a suitable evaluation of their gesture recognition capabilities. An initial attempt by the authors to address this gap was presented in [36], where the concepts of intra- and inter-individual variability in the recognition of hand gestures by XR HMDs were explored. However, the limited sample size, the small set of selected metrics, and the consideration of only a single hand gesture highlighted the need for a significant extension of that work.

## 3. Proposal

In this section, the proposed measurement method for the performance assessment of gesture-based NHMIs implemented within XR HMDs is described. The method builds upon the authors’ previous work in the domain of hands-free NHMIs in XR, particularly involving eye-tracking, head-tracking, and neural interfaces [37,38]. As XR HMDs increasingly support multimodal input, adapting the method to the domain of hands-based interaction contributes to obtaining more comprehensive performance insights into the capabilities of the XR HMDs under evaluation.

The proposed method was developed to ensure consistency and interoperability across different hand gestures and XR HMDs following general ergonomic principles and consistency with common practices in the literature for XR-based target selection tasks:

- *Clear interaction modality*: the gestures representing the human–machine interaction within the XR environment should follow a structured and intuitive process, providing users with a clear and efficient framework for interaction [39]. Common hand gestures should be leveraged, characterized by clear and simple patterns that are easy to learn, thereby promoting a seamless interaction flow [40,41].
- *Accessible XR environment*: the XR environment should feature easily selectable digital icons with high contrast against the background to ensure clear visibility and avoid content overlaps [42]. The design of the XR environment should also accommodate the different hardware and software requirements of off-the-shelf XR HMDs [43]. Prior to each experiment, users should be familiarized with the XR environment and instructed on the gestures to be performed, with feedback provided to clarify correct gesture execution. The sizes, number, and spatial distribution of digital icons should be optimized based on the field of view (FoV) of the XR HMD and the projected depth at which the user is positioned [44]. In fact, as the projected depth between the user and the icons increases, the perceived size of the digital content decreases, corresponding to the FoV’s scaling effect [45]. Additionally, within a given FoV, an increase in the number of icons elevates the perceived spatial density, thereby increasing the likelihood of perceived overlaps [46].
- *Comprehensive performance measurement*: the measurement procedure should be designed to address the variability introduced both within and across individuals, referred to as intra- and inter-individual variability in Section 2, respectively [47]. The measurement procedure should also account for the different acceptance thresholds associated with the gestures under evaluation [48]. Finally, the metrics selected for the performance measurement should reflect the reliability and usability of the gesture-based NHMI developed within the XR HMD considered, and should facilitate comparisons across different gestures and HMDs [49].

### 3.1. Design of the Gesture-Based Interaction

In line with the principle of *clear interaction modality*, the proposed method associates hand gestures with the selection of digital content. As outlined in [50,51], content selection is prioritized over other types of interactions as it represents the first step in identifying the target for subsequent actions. To enhance intuitiveness and usability, a commonly used gesture was leveraged, namely finger-tapping [52], also known as pinch [36], which aligns with natural hand movements and requires minimal cognitive effort [53]. Basically, finger-tapping is a dynamic gesture in which the thumb is brought closer to the index finger.

The finger-tapping interaction was implemented using hand–ray pointing, an interaction technique that defines a ray originating from a reference key point on the hand and extending along a preferred axis. In more detail, the hand’s reference frame is defined as an orthonormal local coordinate system $\left(x_{l}y_{l}z_{l}\right)$, consisting of an origin located at the palm center, not fixed in the world coordinate system but continuously updated in real time based on the current pose of the hand as detected by the tracking system.

In this frame, the $y_{l}$-axis lies along the hand, extending outward from the palm toward the top of the hand. The $x_{l}$-axis lies across the palm, orthogonal to the y-axis and within the palm plane, aligning with the extended thumb, in order to define the local plane of the hand and the $z_{l}$-axis, derived from completing a right-handed orthonormal coordinate system. Although $x_{l}$ and $y_{l}$ axes align with the extended thumb and index finger, respectively, when the hand is open and facing forward, they do not follow the fingers but are instead anchored to the general orientation of the open hand. Therefore, from the origin of the hand frame, the hand ray is defined as a virtual vector extending along the $z_{l}$-axis of the hand’s frame, oriented outward from the palm and continuously updated as the user rotates or tilts their hand. As a result, the user can intuitively direct the hand ray simply by rotating or tilting their wrist, similar to how a laser pointer follows wrist motion. This hand ray is used for pointing and targeting, then allowing the user to interact with virtual content similarly to a laser pointer.

To recognize the gesture, the distance *d* between the fingertip of the thumb and index fingers is compared to an acceptance threshold th: the gesture is correctly recognized when d<th. When the gesture is recognized, the NHMI computes the intersection point between the hand ray and the digital content within the XR environment at that moment.

Although the study considers a single gesture parameterizable in terms of threshold, the proposed method does not preclude the investigation of performance dependence on a broader set of hand gestures.

### 3.2. Design of the XR Environment

**Figure 2:**
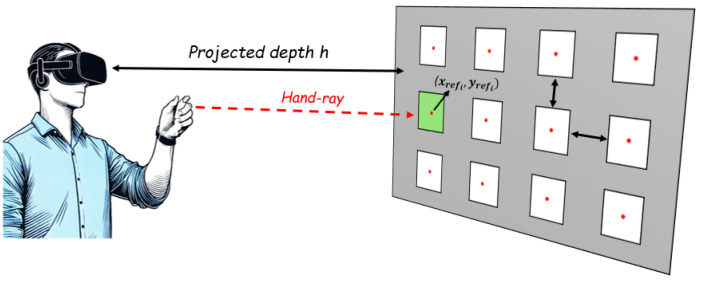
Conceptual design of the proposed XR environment as seen by the user wearing the XR HMD.

According to the principle of *accessible XR environment* described in Section 3, the XR environment was designed to render a set of *N* white icons, intended to be selected by the users by performing the gesture under evaluation. The icons were symmetrically arranged on an x−y plane at a projected depth *h* along the *z*-axis, relative to the user. This x−y plane represents the coordinate system of the digital content, defined in the global coordinate system of the virtual scene, and it is oriented independently of the hand and does not follow the hand’s axes. Each icon was represented as an equal-sized square, with the center marked by a red dot at the coordinates $\left(x_{ref_{i}},y_{ref_{i}}\right)$ for i=1,2,…,N. A graphical representation of the XR environment is shown in Figure 2. For each *i*th icon to be selected, a visual cue appeared on the icon for 2 s, serving as an indicator of the target to be selected, allowing the participant to prepare.

The icon selection occurs in two stages: once the cue disappears, the participant is expected to perform the gesture, which is recognized when the Euclidean distance between thumb tip and the index fingertip becomes smaller than the predefined threshold value for the given scenario (i.e., d<th); at the moment the gesture is recognized, the system computes the intersection point between the hand’s ray and the digital content within the XR environment, denoted as $\left(x_{i},y_{i}\right)$. A selection is considered correct if the computed point $\left(x_{i},y_{i}\right)$ falls within the area of the icon that was previously cued for selection.

To allow users to accurately aim at virtual targets within the scene, the hand ray is displayed through the HMD as a thin ray, similar to a laser pointer, originating from the center of the palm and extending forward along the hand’s z-axis.

Moreover, to allow the users to rest their hands before performing the selection on the next icon, a 2 s resting period was given; in this resting period, users were free to either keep their hand still or relax. The 2 s time interval was chosen in accordance with recent works about NHMIs in XR: in fact, shorter cue times may increase the likelihood of recognition errors, while longer ones tend to slow down the interaction without providing added benefit [54,55,56].

After that, the visual cue was displayed on the next icon in a random sequence, reducing potential bias in the selection process. Once the cues appeared on all icons, the selection task was considered complete. This task was performed for a total of *C* cycles by each user, with a 1-minute break between cycles. This ensured proper assessment of the intra-individual variability. By involving *M* users, each executing *C* cycles of the task, inter-individual variability was also evaluated.

Moreover, to evaluate the performance of the NHMI under varying recognition constraints, different acceptance thresholds th were considered. For each threshold, a corresponding scenario was designed in accordance with the recognition procedure outlined in Section 3.1. Each scenario was executed independently, ensuring that each of the *M* users completed the selection task *C* times for each threshold. To mitigate potential bias and prevent user adaptation to a specific threshold, the scenarios were presented in a randomized order. Additionally, a short resting period was introduced between scenarios to minimize fatigue effects and ensure consistent performance across conditions.

### 3.3. Performance Metrics

In accordance with the third principle (*comprehensive performance measurement*), the performance in gesture recognition was measured by considering the following four metrics, briefly mentioned in Section 1:

- Euclidean distance error (*D*): given the *i*th icon to be selected in a selection cycle, this metric represents the Euclidean distance between the selection point $\left(x_{i},y_{i}\right)$ recognized by the interface and the corresponding reference point $\left(x_{ref_{i}},y_{ref_{i}}\right)$. For each icon, the Euclidean distance error was obtained through (1)
  (1) $$D_{i}=\sqrt{O_{x_{i}}^{2}+O_{y_{i}}^{2}}$$
  where $O_{x_{i}}=x_{i}-x_{ref_{i}}$ and $O_{y_{i}}=y_{i}-y_{ref_{i}}$ are the horizontal and vertical offsets, respectively.
- Selection time (*T*): considering again the *i*th icon in a selection cycle, this metric is defined as the time elapsed between the disappearance of the visual cue and the system’s recognition of the gesture. Hence, it encompasses both the user’s response time to perform the selection and the processing delay of the NHMI in recognizing the gesture.
- Selection accuracy (*A*): for a given selection cycle, denoted as the *j*th, this metric is defined as the ratio of the number of icons correctly recognized as selected by the user ($\hat{N}$) to the total number of icons (*N*) of the *j*th selection cycle.
  (2) $$A_{j}={\left(\frac{\hat{N}}{N}\right)}_{j}\cdot 100$$
- Information transfer rate (ITR): this metric, defined for each selection cycle (denoted again as the *j*th cycle), represents the amount of information that can be conveyed through the NHMI. Expressed in bits per minute, it considers the total number of icons *N* displayed within the XR field of view, the average selection time ${\bar{T}}_{j}$ for the cycle, and the selection accuracy for the cycle, normalized to the range [0,1], expressed as $a_{j}=\frac{A_{j}}{100}$. The ITR for the *j*th cycle is given by Equation (3)
  (3) $$ITR_{j}=\frac{60}{{\bar{T}}_{j}}\cdot \left[log_{2}\left(N\right)+a_{j}\cdot log_{2}\left(a_{j}\right)+\left(1-a_{j}\right)\cdot log_{2}\left(\frac{1-a_{j}}{N-1}\right)\right]$$
  As ITR accounts for misclassification probabilities introduced by the interface, it could offer a more suitable measure for cue-guided selection tasks than Fitts’ Law, which is generally more appropriate for free-form pointing or continuous movement tasks, where selection is assumed to occur without uncertainty [57].

The selection of these performance metrics was motivated by their widespread adoption and recognition as a standard set for evaluating content selection tasks [58,59]. Importantly, this metric set extends beyond those typically used in established hands-free interaction paradigms such as eye-tracking, head-tracking, and Steady-State Visually Evoked Potentials (SSVEPs) [37,38]. Indeed, in such modalities, selection time is not commonly treated as a performance metric; rather, it is usually defined as a fixed parameter within the experimental setup. Additionally, in the context of SSVEP-based interfaces, Euclidean distance is not applicable as such systems do not provide spatial information regarding the user’s gaze position relative to a reference. Instead, they only indicate whether or not a target has been selected.

According to the GUM framework [60], each metric is expressed in terms of a best estimate of (i) its expected value and (ii) the corresponding standard deviation.

### 3.4. Measurement Procedure

For each acceptance threshold of the considered hand gestures, the following operations were conducted:

1. Distance error $D_{j,i}$ and selection time $T_{j,i}$ were assessed for each icon (i=1,2,…,N) within each selection cycle (j=1,2,…,C).
2. For each selection cycle (j=1,2,…,C), the mean Euclidean distance error ${\bar{D}}_{j}$ and the mean selection time ${\bar{T}}_{j}$ across the icons were obtained by means of the arithmetic mean operator. The related standard uncertainties, namely $u(D_{j})$ and $u(T_{j})$, were obtained through a type-A evaluation [60].
3. For each selection cycle (j=1,2,…,C), selection accuracy $A_{j}$ was evaluated through (2). Conversely, the mean value of the ITR, namely ${\bar{ITR}}_{j}$, was obtained through (3). The standard uncertainty of the ITR, namely $u(ITR_{j})$, was obtained by means of the Law of Propagation of Uncertainties (LPU) [60] as follows:
  (4) $$u\left(ITR\right)=\left|{\left.\frac{\partial ITR}{\partial T}\right|}_{T={\bar{T}}_{j}}\cdot u\left(T_{j}\right)\right|$$
4. For each individual (k=1,2,…,M) involved in the experiments, the mean Euclidean distance error ${\bar{D}}^{k}$ and the mean selection time ${\bar{T}}^{k}$ across the cycles were obtained by means of the arithmetic mean operator. The related standard uncertainties, $u(D^{k})$ and $u(T^{k})$, respectively, were obtained through the Law of Total Variance by considering the arithmetic mean of the uncertainty values over each cycle (j=1,2,…,C)), namely $\bar{u(\left[D_{1},D_{2},\ldots ,D_{j},\ldots D_{C}\right])}$ and $\bar{u(\left[T_{1},T_{2},\ldots ,T_{j},\ldots T_{C}\right])}$, along with the standard uncertainty of the mean values over each cycle (j=1,2,…,C)), namely $u(\left[{\bar{D}}_{1},{\bar{D}}_{2},\ldots ,{\bar{D}}_{j},\ldots ,{\bar{D}}_{C}\right])$ and $u(\left[{\bar{T}}_{1},{\bar{T}}_{2},\ldots ,{\bar{T}}_{j},\ldots ,{\bar{T}}_{C}\right])$, according to (5):
  (5) $$\begin{array}{c} u\left(D^{k}\right)=\sqrt{{u(\left[{\bar{D}}_{1},{\bar{D}}_{2},\ldots ,{\bar{D}}_{j},\ldots ,{\bar{D}}_{C}\right])}^{2}+{\bar{u(\left[D_{1},D_{2},\ldots ,D_{j},\ldots D_{C}\right])}}^{2}}\\ u\left(T^{k}\right)=\sqrt{{u(\left[{\bar{T}}_{1},{\bar{T}}_{2},\ldots ,{\bar{T}}_{j},\ldots ,{\bar{T}}_{C}\right])}^{2}+{\bar{u(\left[T_{1},T_{2},\ldots ,T_{j},\ldots T_{C}\right])}}^{2}} \end{array}$$
5. For each individual (k=1,2,…,M), the mean value of selection accuracy ${\bar{A}}^{k}$ across the cycles was obtained by means of the arithmetic mean operator, while the corresponding standard uncertainty $u(A^{k})$ was obtained through a type-A evaluation [60]. Conversely, the mean value of ITR, namely ${\bar{ITR}}^{k}$, was obtained by applying (3) and considering the average selection time across the cycles, namely ${\bar{T}}^{k}$, along with the normalized average selection accuracy across the cycles, namely ${\bar{a}}^{k}={\bar{A}}^{k}/100$. The standard uncertainty $u(ITR^{k})$ was evaluated through the LPU [60] as
  (6) $$u\left(ITR^{k}\right)=\sqrt{{\left({\left.\frac{\partial ITR}{\partial a}\right|}_{a={\bar{a}}^{k}}\cdot u\left(a^{k}\right)\right)}^{2}+{\left({\left.\frac{\partial ITR}{\partial T}\right|}_{T={\bar{T}}^{k}}\cdot u\left(T^{k}\right)\right)}^{2}}$$
6. Once Euclidean distance error, selection time, selection accuracy, and ITR values were obtained for each individual in terms of best estimates of expected value (i.e., the arithmetic means) and standard deviation (i.e., the standard uncertainties), they were aggregated among all the individuals involved in the experiments. In particular, the overall best estimate of the expected value of each metric was obtained as the mean among the arithmetic means of each individual as
  (7) $$\begin{array}{c} \bar{D}=\bar{{\bar{D}}^{1},{\bar{D}}^{2},\ldots ,{\bar{D}}^{k},\ldots ,{\bar{D}}^{M}}\\ \bar{T}=\bar{{\bar{T}}^{1},{\bar{T}}^{2},\ldots ,{\bar{T}}^{k},\ldots ,{\bar{T}}^{M}}\\ \bar{A}=\bar{{\bar{A}}^{1},{\bar{A}}^{2},\ldots ,{\bar{A}}^{k},\ldots ,{\bar{A}}^{M}}\\ \bar{ITR}=\bar{{\bar{ITR}}^{1},{\bar{ITR}}^{2},\ldots ,{\bar{ITR}}^{k},\ldots ,{\bar{ITR}}^{M}} \end{array}$$
  while the overall best estimate of the variance of each metric was obtained through the Law of Total Variance as
  (8) $$\begin{array}{c} u\left(D\right)=\sqrt{u{\left(\left[{\bar{D}}^{1},{\bar{D}}^{2},\ldots ,{\bar{D}}^{k},\ldots ,{\bar{D}}^{M}\right]\right)}^{2}+{\bar{\left[u\left(D^{1}\right),u\left(D^{2}\right),\ldots ,u\left(D^{k}\right),\ldots ,u\left(D^{M}\right)\right]}}^{2}}\\ u\left(T\right)=\sqrt{u{\left(\left[{\bar{T}}^{1},{\bar{T}}^{2},\ldots ,{\bar{T}}^{k},\ldots ,{\bar{T}}^{M}\right]\right)}^{2}+{\bar{\left[u\left(T^{1}\right),u\left(T^{2}\right),\ldots ,u\left(T^{k}\right),\ldots ,u\left(T^{M}\right)\right]}}^{2}}\\ u\left(A\right)=\sqrt{u{\left(\left[{\bar{A}}^{1},{\bar{A}}^{2},\ldots ,{\bar{A}}^{k},\ldots ,{\bar{A}}^{M}\right]\right)}^{2}+{\bar{\left[u\left(A^{1}\right),u\left(A^{2}\right),\ldots ,u\left(A^{k}\right),\ldots ,u\left(A^{M}\right)\right]}}^{2}}\\ u\left(ITR\right)=\sqrt{u{\left(\left[{\bar{ITR}}^{1},{\bar{ITR}}^{2},\ldots ,{\bar{ITR}}^{k},\ldots ,{\bar{ITR}}^{M}\right]\right)}^{2}+{\bar{\left[u\left(ITR^{1}\right),u\left(ITR^{2}\right),\ldots ,u\left(ITR^{k}\right),\ldots ,u\left(ITR^{M}\right)\right]}}^{2}} \end{array}$$
  the first term in the sum represents the inter-individual variability, i.e., the uncertainty of the individual mean values, while the second term represents the intra-individual variability, i.e., the mean of the individual uncertainty values.

## 4. Case Study

Without loss of generality, the proposed method was validated by considering the Microsoft HoloLens 2 [61] as XR HMD. Microsoft HoloLens 2 is an Optical-See-Through (OST) XR HMD, characterized by a diagonal FoV of 52°. As highlighted in Section 1, like other XR manufacturers, Microsoft does not disclose specific details on how data are processed for gesture recognition, only providing general information regarding the sensing modalities, such as the stereoscopic vision system consisting of visible light and IR cameras, along with an IMU that tracks head movement. Despite its unique features, HoloLens 2 is approaching obsolescence. However, the proposed method is designed to be adaptable to any XR HMD, with HoloLens 2 serving merely as a case study to demonstrate its applicability.

A detailed description of the case study, along with the obtained results and the statistical analyses conducted for the performance evaluation, are provided below.

### 4.1. Development of the XR Environment

The XR environment and the NHMI were developed using the Unity game engine, leveraging the *MixedRealityPose* library [62] provided within the *Mixed Reality Toolkit* (MRTK) [63] to access the 3D positions of hand joints. The choice of this specific set of libraries is inherently tied to the choice of using HoloLens 2. However, it is important to note that, had a different XR HMD been used, a corresponding set of compatible libraries would have been adopted. This reflects a general characteristic of XR development, where software components are typically tailored to the target hardware platform.

**Figure 3:**
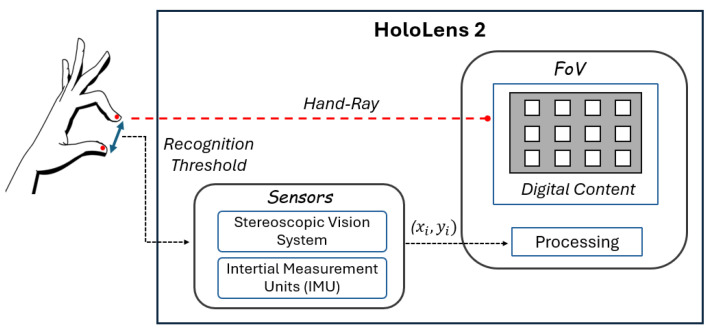
Sketch of the developed gesture-based NHMI.

As illustrated in Figure 3, the XR environment was designed as a 3×4 grid of N=12 square white icons, positioned at a projected depth of h=1.5 m from the user’s perspective. These icons, each sized 0.10×0.10×0.01 m, were symmetrically arranged within the HoloLens FoV, distributed within a reachable and comfortable area in front of the user [45,46], with a spacing of 0.15 m between them along both the x- and y-axes. This design aimed at ensuring usability, visual accessibility, and consistency with the device’s FoV.

For this case study, the depth of the icons was considered negligible in order to isolate and investigate the effects of icon size on the x−y plane. However, the proposed method does not preclude the analysis of depth-related effects and can be extended to scenarios involving cubic icons with non-negligible depth. Moreover, for the specific case study, a single configuration was adopted regarding the number and size of the icons. Nevertheless, the proposed method does not preclude the possibility of repeating the experiments with different icon configurations. This flexibility enables comparative analyses aimed at optimizing system performance.

The visual cue, which indicated the icon to select, was represented by a black cross that appeared on the icon itself. As previously described in Section 3.1 and Section 3.2, the coordinates $\left(x_{i},y_{i}\right)$ selected by the user were determined by the intersection of the hand ray (depicted in Figure 3 as a red dotted line, visible to the user through the HMD) and the grid of icons.

After the execution of exploratory tests, three different recognition thresholds, $th_{r}=1,2,3$ mm, were selected for comparison. Preliminary experiments showed that finger-tapping gestures characterized by threshold values below 1 mm were never recognized; therefore, these threshold values were not practically usable, and only threshold values starting from 1 mm were chosen. For each of the three thresholds, a corresponding scenario ($S_{1}$, $S_{2}$, and $S_{3}$) was implemented to allow users to perform the selection task with a threshold value at a time. As mentioned in Section 3.2, the sequence in which the cue was displayed and the order of the scenarios associated with different thresholds were presented in a randomized order. The task was designed to accommodate both right-handed and left-handed users, ensuring inclusivity and ease of use.

### 4.2. Experimental Setup

The experimental campaign involved M=20 participants, balanced by sex, and aged between 23 and 30 years. Nine of the twenty participants had corrected-to-normal vision, seven had previous experience with XR applications, and three were left-handed. There were no participants with pathologies that could affect reaction times or cause physical and mental fatigue. The experiments were conducted in a dimly lit room where, one at a time, the participants were asked to sit in a comfortable chair, facing a white wall. Lighting conditions were kept uniform and comfortable, with an illuminance level of 600 lux, which is compatible with the recommended range for HoloLens 2 [64].

Before wearing the XR headset, all participants were clearly instructed on the task and asked to perform the finger-tapping gesture in a natural and comfortable way [65]. Participants were instructed to perform gestures with the hand clearly within the gesture frame, and to maintain a consistent forward-facing hand orientation, minimizing occlusion between thumb and index finger during the selection gesture. After that, participants wore the HoloLens 2, adjusting it to display the digital content correctly and carrying out eye calibration prior to starting the experiment in order to guarantee hologram stability [66]. Hence, they were given a brief period, approximately 3–5 min, to familiarize themselves with the system before data collection began.

Hence, for each scenario $S_{th}$ (th=1,2,3 mm), a number of C=5 selection cycles were chosen, each with a different random sequence of the N=12 icons to be selected. As mentioned in Section 3.2, a rest period of one minute was considered at the end of each cycle and each scenario. The entire experiment lasted approximately 40 min for each participant.

A synthetic description of the parameters of the experiment is provided in Table 1.

**Figure 4:**
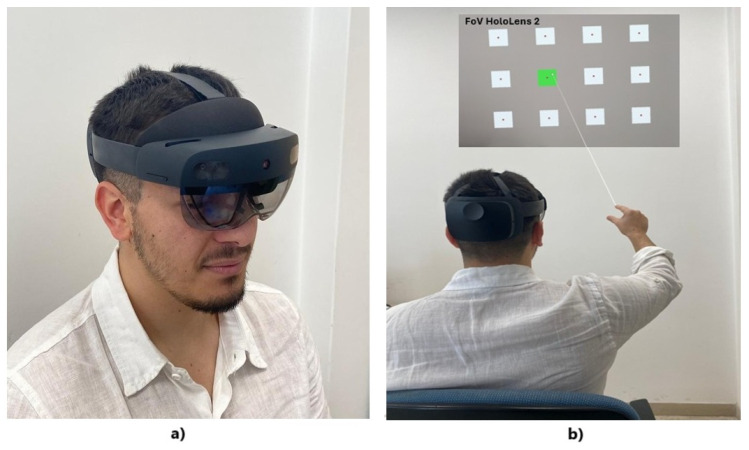
Experimental setup: (**a**) a participant wearing Microsoft HoloLens 2; (**b**) a participant performing the cue-guided selection task.

Finally, the experimental setup is shown in Figure 4, where a participant wearing the HoloLens is shown while performing the cue-guided selection task.

### 4.3. Performance Evaluation

In the case of the 1 mm threshold, HoloLens 2 was not always able to detect the finger-tapping gesture, occasionally preventing users from selecting the desired icons and thus hindering the completion of the cue-guided task. Due to these difficulties, scenario $S_{1}$ was excluded from the analysis.

Table 2 reports the resulting proposed metrics for scenarios $S_{3}$ (th = 3 mm) and $S_{2}$ (th = 2 mm) and for each of the M=20 participants. The values shown in the last row represent the overall mean and uncertainty obtained according to the procedure shown in Section 3.4. All the uncertainties are reported with at most two significant digits.

The overall values of selection accuracy, with a range of approximately [96.0,100.0]% for both scenarios, indicate that the interface adequately recognizes the selections performed by the participants for different thresholds. Although the overall values of the Euclidean distance error vary slightly between the threshold scenarios, the resulting selection time and ITR vary largely. More specifically, in $S_{3}$ scenario, the selection time ranges in the interval [0.7÷2.7] s with an average ITR of 144 bits/min, while, in $S_{2}$ scenario, the mean selection time increases to 3.9 s, reaching a maximum value of 7.7 s and a lower ITR of 64 bits/min.

**Figure 5:**
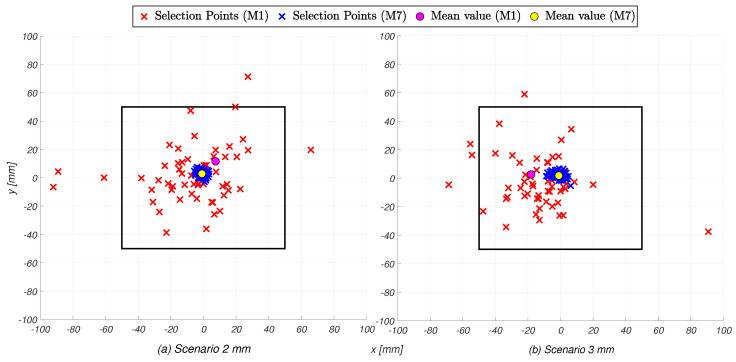
Illustration of the points selected by the highest-performing (blue) and lowest-performing (red) participants, with their mean values (yellow and purple, respectively).

With reference to the results within each threshold scenario, the results presented in the table also reveal potentially significant variability in the Euclidean distance error, both intra- and inter-individually. Figure 5 visually represents this variability. In the left image, corresponding to the $S_{2}$ scenario, the set of selection points are shown for the participant with the lowest average Euclidean distance error (the seventh) and the one with the highest (the first), along with their average values. The same information is depicted in the right image for the $S_{3}$ scenario. Notably, the participants with the best and worst Euclidean distance errors remain the same across both the $S_{2}$ and $S_{3}$ scenarios. This suggests that the intra- and inter-individual variability in content selection are consistent with the choice of different threshold values; that is, participants who tended to perform well (or poorly) under one threshold condition also exhibited similar performance under the other.

**Figure 6:**
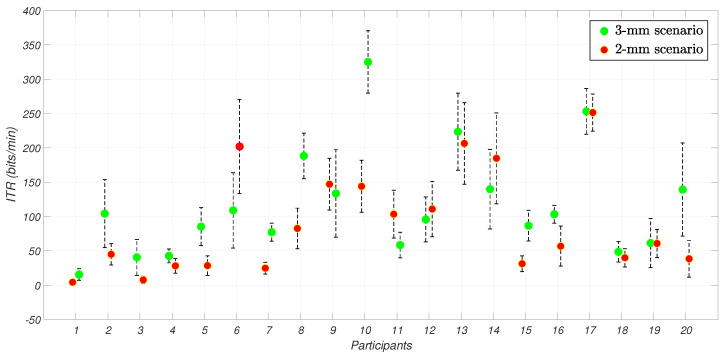
Mean and standard uncertainty for ITR across participants and scenarios. The values were represented according to the order in which the tasks were performed.

By focusing on the amount of conveyed information, the comparison of the resulting ITR for each participant and scenario is shown in Figure 6, where the results are placed in the sequence in which the scenarios were performed. The performance of most participants is comparable between $S_{3}$ and $S_{2}$, underlining a potential consistency in the results among the scenarios.

### 4.4. Statistical Analysis

Appropriate statistical analyses were conducted for a more comprehensive discussion of the obtained results. Due to the non-normality in the individual data distributions, verified by means of the Shapiro–Wilk test (α = 0.05), non-parametric tests were employed [67]. The variability analysis among the $S_{3}$ and $S_{2}$ scenarios was carried out by applying the Kruskal–Wallis test (α = 0.05). By carrying out the test for each metric, the test confirmed the absence of significant differences between scenarios $S_{2}$ and $S_{3}$.

**Figure 7:**
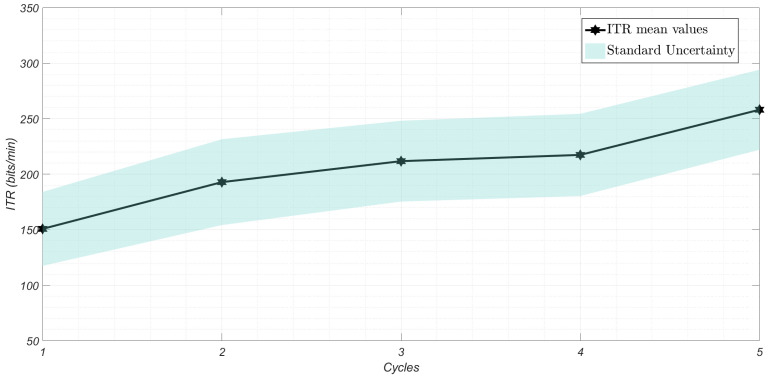
ITR values averaged over 20 participants, with standard uncertainty for each cycle in the 3 mm scenario.

**Figure 8:**
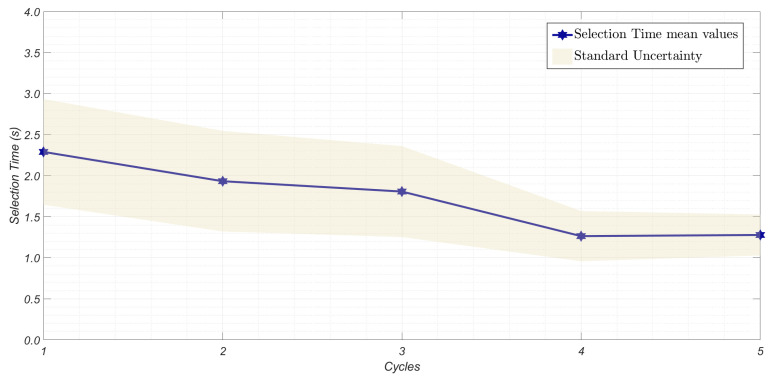
Selection time values averaged over 20 participants, with standard uncertainty for each cycle in the 3 mm scenario.

By considering each scenario separately, intra-individual variability was investigated by comparing, for each metric and each participant, the variability of the results among the C=5 acquisition cycles. As normality conditions were not satisfied, the non-parametric Friedman test was applied (α = 0.05). While for the $S_{2}$ scenario no significant variability between cycles was assessed by the test, the outcomes for $S_{3}$ indicate significant variability in terms of ITR and selection time ($p_{value}$ < 0.01). This variability could be attributed to a more pronounced learning process by the participants over the cycles, as shown in Figure 7 and Figure 8, where the values averaged over all participants for each cycle are represented. As evident, the selection process becomes progressively faster with each cycle, allowing for the transfer of greater amounts of information.

Inter-individual variability was investigated by comparing the results obtained among the different participants. In this case, the non-parametric Kruskal–Wallis test was applied to determine whether there is a statistically significant difference between more than two participants (α = 0.05). The analysis revealed a statistically significant difference between participants in terms of each metric ($p_{value}$ < 0.01), indicating statistically significant variability.

**Figure 9:**
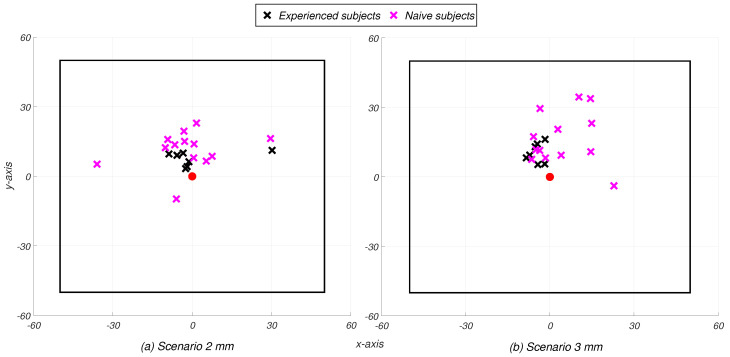
Illustration of the average value of the selection points of each participant, with previous XR experience (in black) and without any XR experience (in violet). The red point represents the origin of the square.

Finally, in order to investigate significant differences in the performance according to the previous experience of each participant, the non-parametric Mann–Whitney test was employed (α = 0.05). For both $S_{2}$ and $S_{3}$ scenarios, the analysis revealed high variability between the two groups only in terms of distance error ($p_{value}<0.01$). This confirms that participants with previous XR experience can achieve higher precision in the task [35] although not necessarily more quickly perform the task. Figure 9 illustrates the average selection points of the participants according to their group within $S_{2}$ and $S_{3}$ scenarios, respectively.

## 5. Conclusions

This paper proposed a method for measuring the performance of gesture-recognition-based Natural Human–Machine Interfaces (NHMIs) implemented within eXtended Reality (XR) Head-Mounted Displays (HMDs). The proposed method follows a systematic approach that ensures compliance with the guidelines of the Guide to the Expression of Uncertainty in Measurement (GUM). The acquired data provide insights into NHMI performance variability, both within individual users over time and across different users. Additionally, the method enables the investigation of learning effects, assessing whether user familiarity with the system influences performance.

To demonstrate the applicability of the proposed method, a case study was conducted using the finger-tapping hand gesture on a Microsoft HoloLens 2 XR HMD. The results showed that the NHMI under test maintained comparable performance across different gesture parameters (i.e., acceptance thresholds). The statistical analyses revealed significant intra-individual variability in the 3 mm threshold condition, where the participants progressively improved their performance over the cycles, suggesting greater adaptability, likely attributable to a learning effect. Furthermore, substantial inter-individual variability was observed, indicating that NHMI performance is influenced not only by gesture parameters but also by individual user characteristics. Additional analysis confirmed that novice participants exhibited the lowest accuracy levels, without necessarily completing the task more quickly.

Overall, the modularity of the proposed approach, as well as relying on input data that are not specific to a single hardware platform, provides a reliable methodological foundation for comparing gesture recognition performance across different XR HMDs, enabling the identification of the most suitable interface that meets the target requirements of the specific application scenarios. As such, the proposed method paves the way for establishing qualification criteria for real-world applications. The metrics obtained under controlled conditions can lead to the definition of performance thresholds, which could be used to assess whether a given interface meets the usability requirements for specific tasks or environments. This would allow favoring the adoption and trustworthiness of XR-based NHMIs in safety- and mission-critical domains.

Finally, to further improve accessibility and corroborate the practical utility of the method, additional research should be devoted to exploring a comparative analysis considering a broader range of gestures (e.g., static vs. dynamic), considering different XR HMDs, and incorporating more diverse user populations (e.g., elderly or mobility-impaired individuals).

## Figures and Tables

**Table 1:** Parameters of the experiment.

No. Icons	Icon Size (m)	Projected Depth (m)	No. Cycles	No. Subjects	No. Scenarios
12	0.10 × 0.10 × 0.01	1.5	5	20	3

**Table 2:** Resulting mean value and standard uncertainty for each metric and each participant.

	th=3 mm				th=2 mm			
Participants	Distance Error (mm)	Selection Time (s)	Accuracy (%)	ITR (bits/min)	Distance Error (mm)	Selection Time (s)	Accuracy (%)	ITR (bits/min)
**#1**	46 (35)	5.7 (3.1)	86.7 (2.0)	16 (8)	39 (24)	20 (11)	86.7 (5.0)	4 (3)
**#2**	13 (3)	1.3 (0.6)	100.0	104 (49)	9 (3)	2.9 (1.0)	98 (2.0)	45 (16)
**#3**	9 (2)	3.4 (2.2)	100.0	41 (26)	26 (25)	15 (10)	95 (3.1)	8 (5)
**#4**	9 (2)	3.3 (0.8)	100.0	43 (10)	26 (44)	4.6 (1.7)	98 (2.0)	28 (11)
**#5**	10 (2)	1.6 (0.5)	100.0	85 (28)	11 (2)	4.9 (2.4)	100.0	29 (14)
**#6**	18 (3)	1.3 (0.6)	100.0	109 (55)	19 (3)	0.7 (0.2)	100.0	202 (69)
**#7**	4 (1)	1.8 (0.3)	100.0	77 (13)	4 (1)	5.6 (2.0)	100.0	25 (9)
**#8**	6 (1)	0.7 (0.1)	100.0	188 (33)	7 (1)	1.7 (0.6)	100.0	83 (30)
**#9**	9 (1)	1.0 (0.5)	100.0	134 (64)	7 (1)	1.0 (0.2)	100.0	147 (38)
**#10**	7 (1)	0.4 (0.1)	100.0	325 (45)	8 (1)	1.0 (0.3)	100.0	144 (38)
**#11**	27 (16)	1.8 (0.4)	91.7 (5.8)	58 (19)	14 (3)	1.3 (0.4)	98 (2.0)	103 (35)
**#12**	25 (5)	1.2 (0.4)	95 (3.1)	96 (33)	18 (4)	1.1 (0.4)	96.7 (2.3)	111 (40)
**#13**	8 (1)	0.6 (0.2)	100.0	223 (56)	8 (1)	0.7 (0.2)	100.0	206 (59)
**#14**	20 (3)	1.0 (0.4)	100.0	140 (58)	15 (3)	0.8 (0.3)	100.0	185 (66)
**#15**	12 (2)	1.6 (0.4)	100.0	87 (22)	16 (4)	4.2 (1.7)	98 (2.0)	31 (11)
**#16**	8 (1)	1.4 (0.2)	100.0	103 (13)	9 (1)	2.5 (1.3)	100.0	57 (29)
**#17**	8 (1)	0.6 (0.1)	100.0	253 (33)	9 (1)	0.6 (0.1)	100.0	251 (27)
**#18**	11 (3)	2.7 (0.8)	98.3 (2.0)	49 (15)	11 (2)	3.5 (1.2)	100.0	40 (13)
**#19**	35 (24)	1.8 (1.0)	93.3 (3.3)	61 (36)	15 (4)	2.0 (0.7)	96.7 (2.3)	61 (21)
**#20**	27 (4)	1.0 (0.5)	100.0	139 (68)	34 (17)	3.0 (2.1)	95 (3.1)	39 (27)
**Average**	16 (11)	1.7 (1.0)	98.2 (2.0)	144 (86)	15 (13)	3.9 (3.8)	98.1 (2.1)	64 (63)

## Data Availability

Data will be available on request.
